# The Impact of Target Frequency on Intra-Individual Variability in Euthymic Bipolar Disorder: A Comparison of Two Sustained Attention Tasks

**DOI:** 10.3389/fpsyt.2016.00106

**Published:** 2016-06-16

**Authors:** Rachel Ann Moss, Andreas Finkelmeyer, Lucy J. Robinson, Jill M. Thompson, Stuart Watson, I. Nicol Ferrier, Peter Gallagher

**Affiliations:** ^1^Institute of Neuroscience, Newcastle upon Tyne, UK; ^2^Northumberland, Tyne and Wear NHS Foundation Trust, Newcastle upon Tyne, UK

**Keywords:** bipolar disorder, attention, neuropsychology, ex-Gaussian distribution, variability

## Abstract

Greater intra-individual variability (IIV) in reaction time (RT) on a sustained attention task has been reported in patients with bipolar disorder (BD) compared with healthy controls. However, it is unclear whether IIV is task specific, or whether it represents general cross-task impairment in BD. This study aimed to investigate whether IIV occurs in sustained attention tasks with different parameters. Twenty-two patients with BD (currently euthymic) and 17 controls completed two sustained attention tasks on different occasions: a low target frequency (~20%) Vigil continuous performance test (CPT) and a high target frequency (~70%) CPT version A-X (CPT-AX). Variability measures (individual standard deviation and coefficient of variation) were calculated per participant, and ex-Gaussian modeling was also applied. This was supplemented by Vincentile analysis to characterize RT distributions. Results indicated that participants (patients and controls) were generally slower and more variable when completing the Vigil CPT compared with CPT-AX. Significant group differences were also observed in the Vigil CPT, with euthymic BD patients being more variable than controls. This result suggests that IIV in BD demonstrates some degree of task specificity. Further research should incorporate analysis of additional RT distributional models (drift diffusion and fast Fourier transform) to fully characterize the pattern of IIV in BD, as well as its relationship to cognitive processes.

## Introduction

Bipolar disorder (BD) is a severe and heterogeneous mood disorder (1). The disorder is associated with marked neurocognitive problems during mood episodes (2, 3), and in full symptomatic recovery. Processing speed, executive functioning, and sustained attention appear to be particularly affected [e.g., Ref. (4–7)]. Indeed, neurocognitive problems are considered a core feature of the disorder (8) and are related to lower quality of life (9). As such, there is interest in developing our understanding of potential cognitive endophenotypes (10), which may have benefits for diagnosis and treatment.

Sustained attention – the ability to maintain concentration over a period of time (11) – is considered a potential cognitive endophenotype of BD (12). To date, studies of sustained attention in BD have tended to rely on global measures of behavioral performance (e.g., overall hits, commission and omission errors). However, there is increasing interest in going beyond these basic measures and examining additional indices of performance such intra-individual variability (IIV).

Attentional IIV refers to extent to which individual reaction time (RT) responses vary during a task (13). It is typically assessed using individual standard deviation (iSD) or the coefficient of variation (CoV), represented by iSD/mean RT (14). IIV may be an informative behavioral marker in BD. Evidence from healthy aging suggests that variability is biologically meaningful; it is heritable (15, 16), is sensitive to the effects of age [i.e., increased IIV with age; (17, 18)], and is a strong behavioral correlate of reduced white matter integrity (19, 20). Furthermore, increased IIV is associated with poorer cognitive functioning (21) and can predict mortality longitudinally (22). IIV may also have clinical utility; for instance, increased IIV is a proposed cognitive marker for prodromal Alzheimer’s disease at the mild cognitive impairment stage (23).

There are statistical and theoretical caveats in simple summary measures of IIV. Indices, such as iSD and CoV, represent pooled data and thus assume that the RT distribution is Gaussian (24). However, empirical RT distributions are typically skewed (25) due to a subset of excessively slow RTs among responses in a normal range (26). A more representative analysis of IIV can be achieved by fitting RT data to an ex-Gaussian distribution (27). In the ex-Gaussian model, the distribution of the RTs is represented as the product (*convolution*) of two randomly distributed variables; one that is Gaussian (normally distributed), and another that is exponential. The latter distribution accounts for the positive skew generally observed in RT distributions (28). The ex-Gaussian distribution is described by three summary parameters; mu (μ) and sigma (σ) (the mean and SD of the Gaussian component), and tau (τ), which references the exponential component.

Conceptually, the distribution of faster responses is indexed by μ and σ. The infrequent, longer RTs which lengthen the tail of the distribution are indexed by τ and can be examined separately from mean RT (24, 29–31).

Analysis of RT distributions using the ex-Gaussian model has been applied successfully in diverse research fields. In general, application of the model has enabled researchers to specify where in a RT distribution groups or individuals differ. Such differences are obscured by examining mean RT in isolation, as excessively slow responding, albeit occasional, can skew this summary statistic (24). Within healthy aging, increases in σ and τ have been reported in older participants compared with a younger sample (29). Indeed, increases in τ may be sensitive to neurodegenerative processes (30). In attention deficit hyperactivity disorder (ADHD), faster overall responding (μ) as well increases in excessively slow responding (τ) has been described (31, 32). Increases in τ observed in ADHD have also been proposed as a candidate endophenotype for the disorder, in part, due to its proposed heritability (33).

Recently, the ex-Gaussian model has been applied in BD (34). The model was used to characterize the RT distribution from a task of sustained attention [Vigil CPT; (35)]. Greater positive skew (τ) in the RT distributions of both euthymic and depressed patients with BD was found – a pattern of responding consistent with the notion of fluctuating attentional task engagement in BD [see also Ref. (36)]. However, the need for replication was also noted in order to determine the influence of factors such as task parameters.

Sustained attention is typically assessed using continuous performance tests [CPTs; (37)]. In the original task, participants responded to an infrequent target “X” and in a later variant, to the “X” only after an “A” cue. Rosvold et al. (37) noted that the ability to correctly classify participants (into those with brain damage and those who were neurologically intact) improved with this increase in task difficulty. Consequently, CPT-AX is considered to be a general marker of brain health (11, 37).

Many CPTs have subsequently been developed, each with numerous procedural variations (38). Common procedural variants include increased/decreased target frequency (39); interstimulus interval (ISI), per block, or adjusted for individual accuracy (39, 40); overall task length, e.g., ranging from 3 min (41) to 30 min (42); and stimulus type, e.g., letters (43, 44) or numbers (45). Finally, the “quality” of the stimuli can be altered through degradation of stimulus integrity over the course of an experiment (46).

It is perhaps not surprising that manipulation in CPT task parameters can affect behavioral response characteristics, some of which are used as markers of the ability to maintain attention. For instance, higher target frequencies are associated with faster mean RTs, as well as increases in errors (47, 48). It is thought that this high target frequency manipulation induces a frequent response tendency (40). Underlying this effect may be an altered demand on response inhibitory and motor control mechanisms (thereby impulsivity), rather than sustained attention *per se* (49). In contrast, low target frequency manipulations result in a slower overall RT (39). Generally, this agrees with the historical definition of vigilance, whereby participants maintain a “vigil” or watch over a long period of time and respond to an infrequent event (50). It has been argued that a low target frequency presentation is therefore a more appropriate index of sustained attention (49). Varying the ISI in a task can also impact response characteristics. A short ISI (<500 ms) is associated with faster mean RTs, as well as increases in omission errors (39). Conversely, a longer ISI is associated with slower RTs, and with increased IIV as measured *via* the ex-Gaussian distribution (40).

As these CPT parameters impact behavioral outcome measures, which may have clinical utility they should be considered when investigating IIV in BD. The aim of this study was therefore to determine whether a similar pattern of IIV would be obtained using a sustained attention task with different parameters in patients with euthymic BD and in healthy controls. The CPT, version AX [CPT-AX; (36)], and the Vigil CPT (35) were utilized. Across both tasks, there are common parameters. Both tasks have a high event rate Parasuraman (50) (Vigil = 64 events/min; CPT-AX = 70 events/min) and both have a similar working memory load (both “1-back” cued target sequences). However, the tasks differ on target frequency. Target sequences are presented infrequently during the Vigil CPT (~20%) compared with CPT-AX (~70%). We predicted that both tasks would result in increased IIV, and with ex-Gaussian modeling, an increase in the τ parameter in patients with BD.

## Materials and Methods

All participants included in the current analyses were from studies conducted within the Institute of Neuroscience at Newcastle University (6, 36). Data included in this study were collected between 2000 and 2003 (Thompson et al.) and 2001 and 2003 (Robinson et al.). Euthymia was confirmed for patients in both studies (see below). These participants were a subset of those reported in Gallagher et al. (34) for whom CPT-AX data were also available.

### Participants

Twenty-two adult euthymic outpatients between the ages of 30 and 57 years (M = 43.13, SD = 7.78) with a SCID (51) confirmed diagnosis of BD were included in the analysis. Clinical interviews were conducted by psychiatrists trained in SCID administration. Recruitment was *via* services within the Northumberland, Tyne and Wear NHS Foundation Trust in the North East of England. Euthymia – defined as a score of ≤7 on the on the 21-item Hamilton Depression Rating Scale [HAMD; (52)] and the Young Mania Rating Scale [YMRS; (53)] – was prospectively verified over 1 month from the initial assessment.

During the verification month, patients completed the Beck Depression Inventory [BDI; (54)] and the Altman Mania Rating Scale [AMRS; (55)] weekly.

All patients were stable and taking psychotropic medication: 16 were prescribed lithium, 10 were prescribed antidepressants, and 5 were prescribed antipsychotics. Exclusion criteria for patients was as follows: (i) presence of another current Axis I diagnosis (except anxiety), (ii) neurological or medical condition, (iii) history of substance or alcohol abuse/dependence over the past 6 months, (iv) prescribed corticosteroids or antihyperintensive medication, and (v) electroconvulsive therapy (ECT) within the past year.

Twenty healthy controls, between the ages of 30 and 53 years (M = 43.55, SD = 6.67) were recruited through local advertisements. Controls did not have a psychiatric history (SCID confirmed) or have a first degree relative with a psychiatric disorder. Groups were well matched on sex, age, and premorbid IQ (National Adult Reading Test, NART) (56) and did not significantly differ in these characteristics (*p* > 0.05; Table 1). All study protocols were approved by the appropriate National Health Service Local Research Ethics Committee. Written informed consent was obtained prior to study participation.

**Table 1:** **Clinical and demographic characteristics of the euthymic BD patients (*n* = 22) and controls (*n* = 20)**.

Variables	Bipolar, mean ± SD	Controls, mean ± SD	*t*/χ^2^	*p*
**Demographic characteristics**
Sex (F:M)	14:8	11:9	0.32	0.569
Age, years	43.13 (7.78)	43.55 (6.67)	0.18	0.855
Premorbid IQ (NART)	111.77 (8.94)	110.65 (7.59)	−0.43	0.665
**Mood ratings**
HAMD (17)	1.68 (1.67)	0.35 (0.67)	–	–
HAMD (21)	1.86 (2.05)	0.35 (0.67)	–	–
**Clinical characteristics**
Age at illness onset, years	25.18 (7.05)	–	*–*	–
Post onset, months	221.22 (98.78)	–	*–*	–

*NART, National Adult Reading Test; HAMD, Hamilton Depression Rating Scale*.

### Neurocognitive Testing: Sustained Attention Tasks

#### Continuous Performance Test, A–X

Single, randomized letters are presented sequentially on a computer screen and participants respond to a target sequence (36). Letters are presented in white, on a black background. In this task, participants respond to target “X,” only when it was presented after an “A” (“AX” target trial). This task uses a stimulus presentation time of 50 ms, and an ISI of 800 ms. In addition, participants respond to an increased number of target sequences.

Over 200 trials of paired stimuli (split into 4 blocks of 50 paired trials), 140 target pairs (35 per block) are presented in 6 min, with no breaks between blocks or practice trials. In both tasks, participants were asked to respond as quickly and accurately.

#### Vigil Continuous Performance Test

In this task, single, randomized letters are presented sequentially on a computer screen for 85 ms, followed by a 900-ms ISI (35). Letters are presented in white, on a black background. Participants respond when they view target “K,” only when cued by an earlier “A” stimulus (“AK” target sequence). Targets occur infrequently in this CPT. Over the course of 480 stimuli, 100 target sequences are presented in 8 min. These targets were pseudo-randomized, so that 25 target sequences are presented in 4 blocks (no breaks given between blocks).

### Data Analysis

First, RTs below 100 ms were removed following established absolute cut-off principles (27, 57), which removed two responses. As the response window for the CPT-AX task could not be extended, the response window for Vigil was restricted to 850 ms.

The restriction was applied to ensure that differences in IIV were not simply due to task-related differences in the time participants had to respond before the next stimulus. A total of 4054 responses were analyzed, and the restriction of the response window removed eight responses (0.19% of total trials). Only correct trials (“hits”) occurring within the response windows were analyzed.

#### RT IIV Analysis

Analysis of RT IIV was applied to correct trials (hits). Common measures of IIV were calculated, which included iSD and CoV (the latter is expressed as a percentage). These measures were applied to all participants (patients *n* = 22, controls *n* = 20). The DISTRIB toolbox (58) in MATLAB^®^ R2013b (59) (The MathsWorks, Inc., Natick, MA, USA) was used to fit the ex-Gaussian probability density function to the distribution of correct RTs.

Three parameters of the ex-Gaussian distribution are estimated per individual using this function; μ, σ, and τ. The algorithm failed to fit the distribution to three control participants, who were then removed from the analysis (patients *n* = 22, controls *n* = 17).

In addition, Vincentile plots were calculated as an overall graphical representation of the data. These plots can be calculated without prior assumptions regarding the theoretical shape of the distribution (60). To calculate Vincentiles, response times per individual are ranked from fastest to slowest into eight bins (each bin presents 12.5% of RTs), and then averaged.

#### Statistical Analysis

Data were analyzed using the Statistical Package for Social Sciences (SPSS), version 21 (61). Matching characteristics for study groups (age, sex, education) were analyzed using independent *t*-tests (continuous variables) and chi-square tests (categorical). Demographic and clinical characteristics were also analyzed between groups in the same manner. Behavioral outcome measures (IIV) were analyzed using repeated measures ANOVA, with task (CPT-AX vs. Vigil) as the within-subjects variable and group (patient vs. controls) as the between-subjects variable.

## Results

### Task

**Figure 1:**
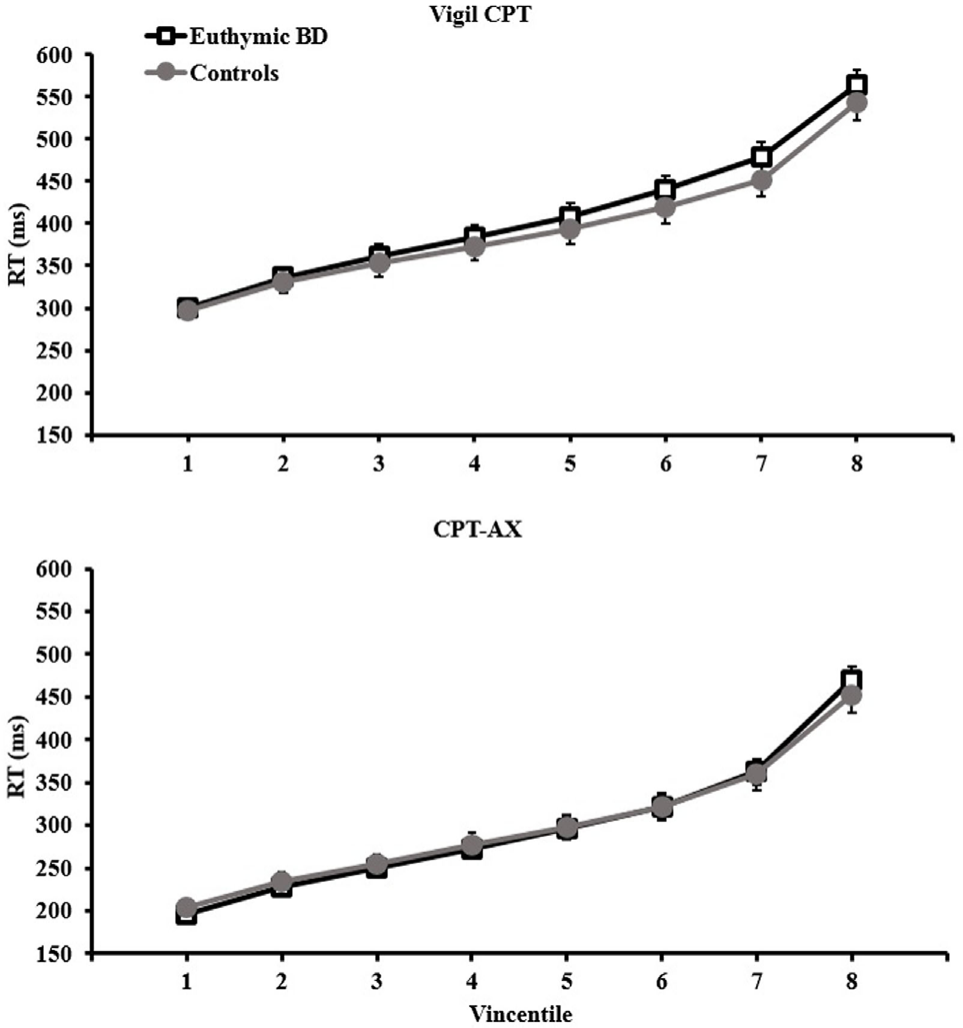
**Vincentile plots (1–8) for euthymic BD (*n* = 22) and controls (*n* = 20) per task**. Mean RTs are taken from the slowest 12.5% (1) to the fastest (8). Plots for Vigil CPT are represented in the *top* panel and CPT-AX in the *bottom*. Error bars represent SEM.

Average response time was slower for the Vigil CPT (mean RT for patients and controls together: M = 394.24, SE = 15.83; ex-Gaussian μ: M = 327.19, SE = 11.11) compared with the CPT-AX (mean RT: M = 299.90, SE = 13.42; ex-Gaussian μ: M = 218.51, SE = 7.32). Examination of the Vincentile plots (Figure 1) indicated that participants performed the Vigil task more slowly across the whole distribution (from *V*_1_ to *V*_8_) compared with CPT-AX (*p* < 0.05). Variability as measured by CoV was higher in the CPT-AX task (M = 26.33, SE = 1.38) compared with Vigil (M = 19.50, SE = 0.98). However, the ex-Gaussian σ parameter was higher in the Vigil task (M = 38.76, SE = 3.40) compared with the CPT-AX (M = 31.23, SE = 2.16). Task had no significant main effect on the remaining IIV parameters (iSD, and ex-Gaussian τ).

### Group

The between-subjects effect for group was significant for ex-Gaussian σ (*p* < 0.001). Euthymic BD patients were more variable overall (ex-Gaussian σ: M = 40.24, SE = 3.26) compared with controls (ex-Gaussian σ: M = 29.75, SE = 3.71). No further between-subjects effects reached significance (see Table 2).

**Table 2:** **Main effects and interactions for each RT parameter from repeated measures ANOVA**.

Effect	*F*(df)	*p*	Partial η^2^
Main effect task (mean RT)	226.86_(1,40)_	0.00***	0.85
Main effect task (iSD)	1.77_(1,40)_	0.19	0.04
Main effect task (CoV)	107.60_(1,40)_	0.00***	0.72
Main effect task (ex-Gaussian μ)	200.43_(1,37)_	0.00***	0.84
Main effect task (ex-Gaussian σ)	6.96_(1,37)_	0.01**	0.15
Main effect task (ex-Gaussian τ)	3.60_(1,37)_	0.06	0.08
Main effect diagnosis (mean RT)	0.16_(1,40)_	0.69	0.00
Main effect diagnosis (iSD)	2.13_(1,40)_	0.15	0.05
Main effect diagnosis (CoV)	2.66_(1,40)_	0.11	0.06
Main effect diagnosis (ex-Gaussian μ)	1.15_(1,37)_	0.28	0.03
Main effect diagnosis (ex-Gaussian σ)	4.50_(1,37)_	0.04*	0.10
Main effect diagnosis (ex-Gaussian τ)	0.06_(1,37)_	0.79	0.00
Task × diagnosis (mean RT)	1.21_(1,40)_	0.27	0.29
Task × diagnosis (iSD)	0.47_(1,40)_	0.49	0.01
Task × diagnosis (CoV)	2.49_(1,40)_	0.12	0.05
Task × diagnosis (ex-Gaussian μ)	3.86_(1,37)_	0.06	0.09
Task × diagnosis (ex-Gaussian σ)	5.46_(1,37)_	0.02*	0.12
Task × diagnosis (ex-Gaussian τ)	2.32_(1,37)_	0.13	0.05

*RT, reaction time; iSD, individual standard deviation; CoV, coefficient of variation; η^2^, eta-squared*.

Significant results are highlighted in bold, *p < 0.05, **p < 0.01, ***p < 0.001.

### Task × Group Interaction

**Figure 2:**
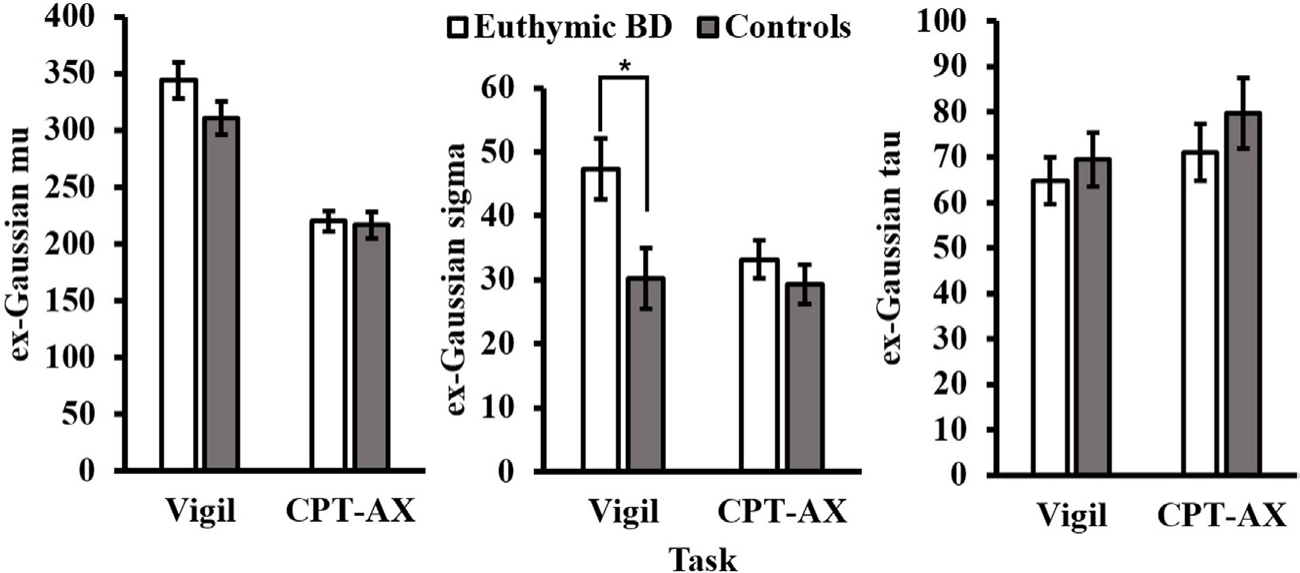
**Ex-Gaussian parameters for euthymic BD and controls per task (CPT-AX and Vigil)**. Euthymic BD patients were more variable than controls, as indicated by ex-Gaussian σ (*p < 0.05), but only in the Vigil CPT. Error bar represent SEM.

A significant interaction was observed between task and diagnosis for ex-Gaussian σ (*p* < 0.05; Table 2). The interaction was driven by differences in variability between the groups in the Vigil task [*t*_(37)_ = −2.51, *p* < 0.05], but not the CPT-AX [*t*_(37)_ = −0.882, *p* > 0.05]. Here, bipolar euthymic patients were more variable (M = 47.34, SE = 4.74) than controls (M = 30.18, SE = 4.73) (Figure 2). Results were also assessed separately per task, between patients and controls.

### Exploratory Analyses

For the Vigil and CPT-AX tasks, exploratory two-tailed Spearman’s correlations were performed between IIV variables (iSD, CoV, and ex-Gaussian parameters), clinical characteristics (HAMD_21_, months post onset, and age of onset), and demographic (age) for patients. Demographic information (age) was also investigated in controls. No significant correlations were observed between IIV indices and any clinical or demographic characteristic in either task (all *p* > 0.05; see Table S1 for Vigil CPT and Table S2 for CPT-AX in Supplementary Material) for patients.

Age did not significantly correlate (all *p* > 0.05) with any IIV index in either task for controls (see Table S3 in Supplementary Material).

## Discussion

This study investigated RT IIV during two differing sustained attention tasks (CPT-AX and Vigil CPT) in euthymic BD patients and healthy controls. The sample overall (patients and controls) completed the low target frequency Vigil CPT more slowly than the high target frequency CPT-AX, as indicated by greater values of mean RT and ex-Gaussian μ. In addition, variability was higher for sample overall for ex-Gaussian σ, but only in the Vigil CPT. Variability as measured by iSD and ex-Gaussian τ between each task was similar for the sample overall. Between groups, however, euthymic BD patients exhibited greater values of σ compared with controls, but only during the Vigil CPT. All other indices of variability (iSD, CoV, and ex-Gaussian τ) were similar between patients and controls in the CPT-AX and Vigil.

The variability in responding demonstrated by euthymic BD patients suggests irregular attentional engagement. However, this inconsistency is only observed under the unique task conditions of the Vigil CPT. In Parasuraman’s (50) definition, both tasks included in this study had high event rates (more than 60 stimuli presented per minute). Such tasks tend to be taxing, resulting in a vigilance decrement (i.e., reduction in accuracy over time). The high event rate and low target frequency parameters of the Vigil CPT may have resulted in unique task conditions requiring more effortful processing. This level of processing would be required to maintain an adequate level of attentional task engagement in response to increased task demands (62, 63). It is possible that the euthymic BD patients in this study were more sensitive to the task conditions induced during the Vigil CPT, reflected by the greater variability in responding.

Working memory load should also be considered. It should be noted that CPTs generally involve a modest contribution of working memory, namely, goal maintenance (6, 36). However, as both tasks required participants to maintain a target sequence of equal length (“AX” for the CPT-AX and “AK” for the Vigil CPT), the contribution of working memory was controlled for. While target percentage was the primary interest of this study, other differences between the tasks, such as stimulus presentation and ISI may also have influenced results.

The results of this study may fit within an accelerated cognitive aging interpretation of disease progression in BD (64, 65), as increased IIV is associated with age-related processes (17, 18). The accelerated aging model highlights that many of the changes observed in BD such as altered brain structure, and cognitive impairments among others, mirror those observed in healthy aging (66).

Methodological considerations of the study should also be taken into account. In the data set analyzed in this study, we did not observe increased ex-Gaussian τ in the Vigil CPT in patients. We included a subsample of euthymic bipolar patients (*n* = 22) and controls (*n* = 17) from the larger sample (*n* = 86 per group) in Gallagher et al. (34), who completed both the Vigil CPT and the CPT-AX. It is possible that the results of this study may have been a cohort effect from using this subsample from the larger study, given that the observed increase in σ was only observed in BD depression, not in euthymic patients in the larger study. It is also possible that the results of this study were due to difference in the demographic and/or clinical characteristics between the samples. However, this is unlikely, given similar reported characteristics between the two studies (age, IQ, depression severity), as well as lack of relationship between these characteristics and any RT IIV parameters. As such, it is likely that the lack of comparison between the results of this study, and those of Gallagher et al. (34) may be due to smaller sample size, as opposed to study characteristics. The small sample size of this study can be considered a limitation.

It should be noted that a longer window for correct responses was included in the Gallagher et al. ex-Gaussian modeling of Vigil CPT responses. In addition to the full response window being used (985 ms), “late” responses of up to 1970 ms were included under certain circumstances.

This window was restricted in this study to 850 ms, which may have resulted in a shift within the fitted ex-Gaussian distributional parameters. However, in the present subsample, this only resulted in a very small number of responses that were excluded and thus is unlikely to have resulted in a large change in the group results. In Table S4 in Supplementary Material, we investigated the possibility that use of a restricted response window removed data contributing to positive skew (τ). Extension of the response window did not alter the results from analysis of the Vigil CPT, between patients and controls – ex-Gaussian σ remained the sole significant between-group difference.

Based on the results of this study, future research should clarify the role that other task parameters have upon RT distributions. CPT procedural variations, such as event rate, have also been shown to impact on mean RTs (39, 50, 67). Identifying the independent contributions of each task parameter (e.g., such as speed of stimulus presentation, working memory load, etc.) would be worthwhile, as CPTs generally manipulate more than one parameter. For instance, the CPT variants included in this study both used high event rates, yet varied on target frequency. Future work should clarify the conditions necessary for task-dependent variability in RT distributions.

A further point of discussion concerns the interpretation of the ex-Gaussian model. While authors have suggested that certain components of the model represent specific cognitive functions [e.g., increases in τ and attentional lapses; (28)], caution in utilizing this interpretation is warranted. As the model lacks solid theoretical underpinning, application of the ex-Gaussian model should be considered descriptive (68). Consequently, use of this model in isolation cannot account for the cognitive factors that drive behavioral performance (27, 69).

With these caveats considered, future research could combine theoretical RT models with ex-Gaussian distribution fitting. One candidate is the drift diffusion model (DDM) (70, 71). In brief, the model assumes that responses are made following accumulation of information that reaches a threshold (correct or incorrect decision boundary). After this threshold is reached, the participant responds. The model consists of three parameters; (i) drift rate (*v*), which is the rate of information acquired from a stimulus to make a response decision; (ii) boundary separation (*a*), which contains information about response biases (i.e., speed/accuracy trade-offs), and (iii) non-decision time (*Ter*), which indexes other processes that are different from decision-making (e.g., motor preparation). Combining ex-Gaussian analysis with the DDM may serve to strengthen the link between altered response profiles observed in psychiatric populations (such as BD), and underlying cognitive factors (e.g., decision processes), which may account for such differences.

Further research could also combine analysis of the ex-Gaussian distribution with examining the temporal components of RT IIV. With use of the Fast Fourier Transform (FFT), periodic patterns of responding that are specific to certain time scales (temporal frequency bands) can be detected; manifesting itself as peaks in the (spectral) power at the specific frequency band (72). Interestingly, analysis of FFT may indicate underlying abnormalities, such as inefficient processing in specific neural and/or resting-state networks [e.g., as used in ADHD; (31, 72)]. As altered network activity has been reported frequently in BD (73), analysis of the oscillatory pattern of response times warrants further investigation. This may shed light on the underlying pathophysiology associated with the disorder, which may provide novel targets for psychopharmacological interventions.

To conclude, while all participants were slower and more variable when completing the Vigil CPT compared with the CPT-AX, euthymic BD patients exhibited greater variability (σ) than controls in the Vigil CPT. In addition, the results also suggest IIV has a degree of task specificity. Future research should consider alternative RT models and analyses of temporal instability of RT concurrently with the ex-Gaussian.

## Author Contributions

Data collection: LR, JT, SW, IF, and PG. Data analysis: RM and AF. Manuscript writing: RM, AF, LR, JT, SW, IF, and PG.

## Conflict of Interest Statement

The authors declare that the research was conducted in the absence of any commercial or financial relationships that could be construed as a potential conflict of interest.
