# Supplementary material for: The Impact of Target Frequency on Intra-Individual Variability in Euthymic Bipolar Disorder: A Comparison of Two Sustained Attention Tasks
Source: Front Psychiatry. 2016 Jun 16;7:106. doi: 10.3389/fpsyt.2016.00106 (PMC4909748; doi:10.3389/fpsyt.2016.00106)
Supplement: Supplementary file 2 [file Table_2.DOCX]

Table 2. Correlations between RT and IIV indices (including ex-Gaussian parameters), and clinical characteristics for the CPT-AX (patients only, *n* = 22).

| **Parameter** | **Age** | **Age of onset (years)** | **Months post onset (years)** | **HAMD_21_** |
| --- | --- | --- | --- | --- |
| mean RT | -0.08 | -0.04 | -0.03 | -0.22 |
| iSD | 0.12 | -0.10 | 0.14 | -0.04 |
| CoV | 0.19 | 0.01 | 0.18 | 0.04 |
| ex-Gaussian mu | -0.19 | 0.02 | -0.22 | -0.15 |
| ex-Gaussian sigma | -0.02 | 0.22 | -0.12 | 0.26 |
| ex-Gaussian tau | 0.03 | -0.00 | 0.13 | -0.06 |

*Note.* RT = Reaction Time, iSD = individual Standard Deviation; CoV = Coefficient of Variation, HAMD_21_ = Hamilton Depression Scale, 21- item.
