# Supplementary material for: The Impact of Target Frequency on Intra-Individual Variability in Euthymic Bipolar Disorder: A Comparison of Two Sustained Attention Tasks
Source: Front Psychiatry. 2016 Jun 16;7:106. doi: 10.3389/fpsyt.2016.00106 (PMC4909748; doi:10.3389/fpsyt.2016.00106)
Supplement: Supplementary file 3 [file Table_3.DOCX]

Table 3. Correlations between RT and IIV indices (including ex-Gaussian parameters), and age for the Vigil CPT and CPT-AX in controls.

| **Parameter** | **Vigil CPT** | **CPT-AX** |
| --- | --- | --- |
| mean RT^a^ | 0.05 | 0.02 |
| iSD | -0.02 | 0.07 |
| CoV | 0.06 | 0.01 |
| Ex-Gaussian mu^bc^ | -0.05 | -0.05 |
| Ex-Gaussian sigma | -0.32 | -0.16 |
| Ex-Gaussian tau | 0.18 | 0.11 |

^a^ *n* for mean RT, iSD, and CoV is 20 for Vigil CPT and CPT-AX.

^b^ *n* for ex-Gaussian mu, sigma, and tau for Vigil CPT is 19.

^c^ *n* for ex-Gaussian mu, sigma, and tau for CPT-AX is 18.
