# Supplementary material for: The Impact of Target Frequency on Intra-Individual Variability in Euthymic Bipolar Disorder: A Comparison of Two Sustained Attention Tasks
Source: Front Psychiatry. 2016 Jun 16;7:106. doi: 10.3389/fpsyt.2016.00106 (PMC4909748; doi:10.3389/fpsyt.2016.00106)
Supplement: Supplementary file 4 [file Table_4.DOCX]

Table 4. Main effects and interactions for each reaction time parameter using the extended Vigil window (1970 ms) from repeated measures ANOVA.

| **Effect** | ***F(df)*** | ***p*** | ***Partial Ƞ^2^*** |
| --- | --- | --- | --- |
| Main effect Task (mean RT) | 62.48 _(1, 40)_ | **0.00***** | 0.61 |
| Main effect Task (iSD) | 1.26 _(1, 40)_ | 0.26 | 0.03 |
| Main effect Task (CoV) | 47.33 _(1, 40)_ | **0.00***** | 0.54 |
| Main effect Task (ex-Gaussian mu) | 196.68 _(37)_ | **0.00***** | 0.84 |
| Main effect Task (ex-Gaussian sigma) | 7.57 _(37)_ | **0.00**** | 0.17 |
| Main effect Task (ex-Gaussian tau) | 4.81 _(37)_ | **0.03*** | 0.11 |
|  |  |  |  |
| Main effect Diagnosis (mean RT) | 0.24 _(1, 40)_ | 0.62 | 0.00 |
| Main effect Diagnosis (iSD) | 2.37 _(1, 40)_ | 0.13 | 0.05 |
| Main effect Diagnosis (CoV) | 3.41 _(1, 40)_ | 0.07 | 0.07 |
| Main effect Diagnosis (ex-Gaussian mu) | 1.10 _(37)_ | 0.29 | 0.02 |
| Main effect Diagnosis (ex-Gaussian sigma) | 4.35 _(37)_ | **0.04*** | 0.10 |
| Main effect Diagnosis (ex-Gaussian tau) | 0.11 _(37)_ | 0.74 | 0.00 |
|  |  |  |  |
| Task x Diagnosis (mean RT) | 0.32 _(1, 40)_ | 0.57 | 0.00 |
| Task x Diagnosis (iSD) | 0.46 _(1, 40)_ | 0.49 | 0.01 |
| Task x Diagnosis (CoV) | 1.59 _(1, 40)_ | 0.21 | 0.03 |
| Task x Diagnosis (ex-Gaussian mu) | 3.61 _(37)_ | 0.06 | 0.08 |
| Task x Diagnosis (ex-Gaussian sigma) | 5.19 _(37)_ | **0.02*** | 0.12 |
| Task x Diagnosis (ex-Gaussian tau) | 1.82 _(37)_ | 0.18 | 0.04 |

*Note.* RT = Reaction Time; iSD = Individual standard deviation; CoV = Coefficient of Variation; *Ƞ^2^*= Eta-squared.

**p* < 0.05 ***p* < 0.01 *** *p* < 0.001
